# The mediatıng role of insomnia in the effect of depression on healthy lifestyle behaviors in post-menopausal women

**DOI:** 10.1590/1806-9282.20240943

**Published:** 2024-12-02

**Authors:** Mehmet Ali Sen, Eda Yakıt Ak

**Affiliations:** 1 Dicle University, Atatürk Vocational School of Health Services – Diyarbakır, Turkey.

## INTRODUCTION

Menopausal women frequently report both physical and psychological manifestations attributed to fluctuations in estrogen levels and diminished ovarian function. Research by Zhou et al. indicates that approximately two-thirds of women undergo these symptoms during the menopausal transition^1^. Additionally, psychiatric symptoms such as anxiety, depression, and sexual dysfunction as well as genito-urinary syndrome are commonly observed. Sleep disturbances, in particular, are highly prevalent in women undergoing this phase of life. Within the existing literature, reported rates of sleep difficulties among menopausal women vary between 39 and 60%^2,3^. Many factors caused by hormonal changes, such as increased irritability, hot flushes, and increased need to urinate at night, lead to sleep problems^4,5^.

Persistent and severe sleep disturbances can significantly jeopardize the physical and mental well-being of women^6^. It is established that depression and sleep disorders share a close interconnection^7^. Research has consistently highlighted the association between depression and sleep issues among menopausal women^8,9^. Factors influencing sleep quality encompass age, a familial history of insomnia, occurrences of insomnia episodes, stress levels, overall health status, the presence of chronic pain, vasomotor symptoms, and neuropsychiatric manifestations^1,10^.

Sleep disruption in menopause serves as more than just a symptom of depression; it also constitutes a potential precursor to future depressive episodes. A heightened comprehension of the underlying mechanisms contributing to insomnia’s onset and persistence during this transitional phase will prove instrumental in formulating targeted treatment approaches. Grasping the origins of sleep disturbances in post-menopausal women and implementing efficacious preventative and therapeutic interventions holds paramount significance for enhancing psychological well-being and overall healthy lifestyle behaviors. There are no studies in the literature that examine the relationship between these three conditions experienced by menopausal women in depth. The results of this study will contribute to a more conceptual understanding of the relationship between sleep problems, depression, and the healthy lifestyle of menopausal women.

This study was conducted to determine the mediating role of insomnia in the effect of depression on healthy lifestyle behaviors in post-menopausal women.

## METHODS

This descriptive, cross-sectional study was conducted via an online survey between October 15, 2022, and July 30, 2023. We targeted Turkish-speaking women over 45 years of age who had not menstruated for at least 1 year and who spoke Turkish. We collected data through social media (Linkedin, Instagram, and Facebook). In the questionnaire, women were asked how long they had been completely menstrually absent. This question allowed us to identify pre-menopausal and menopausal women. Women who reported that they had not menstruated for less than 1 year were excluded from the survey. Women under 45 years of age who participated in the online survey and did not answer all questions were also excluded. The power of the sample to represent the population was calculated using the G-power program. The correlation value in the article of Humeniuk et al. was taken as the main parameter in the study, and the sample size required for 95% power at an effect size of d=0.95 was calculated as at least n=151. In pursuit of maximizing diversity within the study, it was ultimately completed with a cohort of 407 women. Ethical approval for the study and institutional approval were obtained (E-14679147-663.05-391979). Descriptive Information Form, Women’s Health Initiative Insomnia Scale (WHIIS), Beck Depression Inventory (BDI), and Healthy Lifestyle Behaviors Scale II (HLBS II) were used as data collection tools.

The Women’s Health Initiative Insomnia Scale-WHIIS is a Likert-type scale consisting of five questions. The highest score on the scale indicates the greatest degree of insomnia symptoms. Scores between 0 and 20 are obtained from the scale in total, and a total score of 10 and above indicates that women have insomnia^11,12^. While the Cronbach alpha value in the Turkish validation study was 0.85, this value was 0.81 in our study.

The Beck Depression Inventory (BDI) consists of 21 questions and a total score of 0–63, with higher scores indicating more severe depressive symptoms. The level of depression is determined by summing the number of scores obtained: lack of depression or low mood (0–10 scores), moderate depression (11–27 scores), and severe depression (28 scores or more)^13,14^. While the Cronbach alpha value in the Turkish validation study was 0.80, this value was 0.91 in our study.

Healthy Lifestyle Behaviors Scale II (HLBS II): The scale evaluates the behaviors associated with the individual’s healthy lifestyle. The scale consists of a total of 52 items and six sub-dimensions. The subscales are spiritual development, health responsibility, physical activity, nutrition, interpersonal relationships, and stress management. The minimum score for the whole scale is 52, and the maximum score is 208^15,16^. While the Cronbach alpha value in the Turkish validation study was 0.92, this value was 0.92 in our study.

Data were analyzed using Statistical Package for the Social Sciences (SPSS) 21.0 and a 95% confidence level. While the t-test was used to analyze demographic variables with two groups, the ANOVA test was used in the analysis of variables with k (k>2) groups. Groups with differences were analyzed with the Tukey test. In addition, SPSS PROCESS macro 4 regression analysis was used for the mediation factor.

## RESULTS

The mean age of the participants was 52.99±8.62 years; 38.6% (n=157) were 45–48 years old, 31.4% (n=128) were 49–54 years old, and 30% (n=122) were 55 years old or older; 41.2% (n=162) were illiterate, 36.9% (n=150) were literate or primary school graduates, and 21.9% (n=89) were secondary school graduates or above; 34.2% (n=139) of the participants had a poor income, 54.7% (n=234) had a medium income, and 8.4% (n=34) had a good income.

A significant correlation was found between the WHIIS, BDI, and HLBS II scales and the variables of daily sleep hours (p=0.000), self-reported insomnia (p=0.000), past deterioration in sleep quality (p=0.000), and perceived frequency of insomnia (p=0.000). The comparison of women’s sleep status characteristics with WHIIS, BDI, and HLBS II scales is shown in Table 1.

**Table 1:** Comparison of women’s sleep characteristics with Beck Depression Inventory and Healthy Lifestyle Behaviors Scale II scale scores.

Variables (n=407)			BDI	HLBS II Total score	HLBS II Health responsibility	HLBS II Physical activity	HLBS II Nutrition	HLBS II Spiritual development	HLBS II Interpersonal relationships	HLBS II Stress management
	n	%	± SD	± SD	± SD	± SD	± SD	± SD	± SD	± SD
Daily sleep hours										
3–5 h	138	33.9	20.05±10.14^a^	111.09±16.74^a^	18.91±4.05	14.03±4.45	19.84±3.50	20.80±3.94^b^	21.0±3.79^b^	16.51±3.68^b^
6–7 h	177	43.5	16.44±9.76^b^	114.32±19.51^b^	19.05±4.54	13.47±4.06	20.59±3.91	22.22±4.52^a^	22.12±4.11^a^	16.87±4.30
8–10 h	92	22.6	9.59±9.15^a,b^	119.11±20.29^a,b^	19.64±4.75	14.75±4.31	20.77±3.80	22.88±4.42^a^	23.09±4.45^a^	17.97±4.17^a^
F			31.887	5.015	0.810	2.757	2.194	7.330	7.443	3.744
P			**0.000**	**0.000**	0.446	0.065	0.113	**0.001**	**0.001**	**0.002**
Experiencing insomnia										
No	158	38.8	11.24±9.71	119.59±20.82	19.70±4.77	14.62±4.37	21.26±4.01	22.94±4.49	23.10±4.33	17.97±4.36
Yes	249	61.2	19.21±9.76	110.96±16.93	18.78±4.16	13.53±4.15	19.81±3.50	21.22±4.18	21.24±3.88	16.38±3.82
T			-9.042	4.576	2.067	2.535	3.833	3.938	4.492	3.865
P			**0.000**	**0.000**	**0.039**	**0.012**	**0.000**	**0.000**	**0.000**	**0.000**
Deterioration in sleep quality compared to the past										
No	105	25.8	11.55±10.35	120.12±20.01	19.70±4.55	15.17±4.03	21.05±3.87	22.70±4.87	23.06±4.67	18.46±4.33
Yes	302	74.2	17.70±10.06	112.29±18.22	18.94±4.38	13.53±4.28	20.14±3.70	21.61±4.16	21.58±3.89	16.49±3.90
T			-5.354	3.699	1.507	3.445	2.131	2.201	3.176	4.320
P			**0.000**	**0.000**	0.133	**0.001**	**0.034**	**0.028**	**0.002**	**0.000**
Insomnia frequency										
Every day	112	27.5	21.08±10.14^a^	110.05±15.97^b^	18.51±4.03	13.64±4.19	19.35±3.20^b^	20.98±4.18^b^	21.26±3.65^b^	16.30±3.76^b^
Twice or more per week	121	29.7	16.99±9.30	111.64±16.95^b^	19.15±4.48	14.56±4.47	20.92±4.03	22.57±4.30	22.56±4.35	17.99±4.26^b^
Once a week	122	30.0	13.14±9.50	117.74±19.60	19.08±4.19	13.27±3.69	20.21±3.44	21.46±4.12^a^	21.60±3.99	16.02±3.37^b^
Never	52	12.8	10.37±11.01^b^	121.65±24.23^a^	20.58±5.38	14.77±4.97	21.69±4.39^a^	23.27±5.01^a^	22.92±4.78^a^	18.42±5.10^a^
F			19.514	6.881	2.620	2.701	5.985	4.831	3.201	8.223
P			**0.000**	**0.000**	0.050	0.045	**0.001**	**0.003**	**0.003**	**0.000**

^a, b^groups where there is a difference. t: Student’s t-test; WHIIS: Women’s Health Initiative Insomnia Scale; BDI: Beck Depression Inventory; HLBS II: Healthy Lifestyle Behaviors Scale II; SD: standard deviation. Bold indicates p<0.05.

The mean WHIIS total score, BDI total score, and HLBSS II total score of the women were 8.23±4.75, 16.12±10.48, and 114.31±18.99, respectively. There is a positive relationship between the WHIIS score and BDI total score (r=0.477) and a negative relationship between the WHIIS score and HLBSS total score (r=-0.210) (p<0.01, p<0.05) (Table 2).

**Table 2:** The relationship between Women’s Health Initiative Insomnia Scale, Beck Depression Inventory, and Healthy Lifestyle Behaviors Scale II total and subfactor scores of women.

Scales (n=407)	Score range available	± SD	Score range received		1.	2.
1. WHIIS total score	0–20	8.23±4.75	0–20	**r**	**1**	
				**p**		
2. BDI Total score	0–63	16.12±10.48	0–63	**r**	0.477**	1
				**p**	**0.000**	
3. HLBS II Total score	52–208	114.31±18.99	72–170	**r**	-0.210**	-0.194**
				**p**	**0.000**	**0.000**
3.1. HLBS II Health responsibility	9–36	19.14±4.43	9–33	**r**	-0.097	-0.020
				**p**	0.051	0.684
	8–32	13.95±4.27	8–25	**r**	-0.158**	0.025
				**p**	**0.001**	0.617
3.2. HLBS II Physical activity	9–36	20.38±3.77	13–33	**r**	-0.155**	-0.145**
				**p**	**0.002**	**0.003**
3.3. HLBS II Nutrition	9–36	21.89±4.38	12–33	**r**	-0.101*	-0.307**
				**p**	**0.042**	**0.000**
3.4. HLBS II Spiritual development	9–36	21.96±4.15	12–36	**r**	-0.170**	-0.288**
				**p**	**0.001**	**0.000**
3.5. HLBS II Interpersonal relationships	8–32	17.00±4.11	8–30	r	-0.280**	-0.150**
				p	0.000	0.002
	$\bar{X}$ **± SS**	**WHIIS**	**BDI**		**HLBS II**	
Age	53.00±8.62	r=0.175**	r=0.088		r=-0.151**	
		**p=0.000**	p=0.075		**p=0.002**	
Year of marriage	30.48±10.07	r=0.091	r=0.175**		r=-0.124*	
		p=0.067	**p=0.000**		**p=0.012**	

X: mean; SD: standard deviation; r: correlation coefficient; F: one way Anova; t: Student’s t-test; WHIIS: Women’s Health Initiative Insomnia Scale; BDI: Beck Depression Inventory; HLBS II: Healthy Lifestyle Behaviors Scale II; SD: standard deviation..

*p<0.05;

**p<0.01. Bold indicates p<0.05.

**Figure 1:**
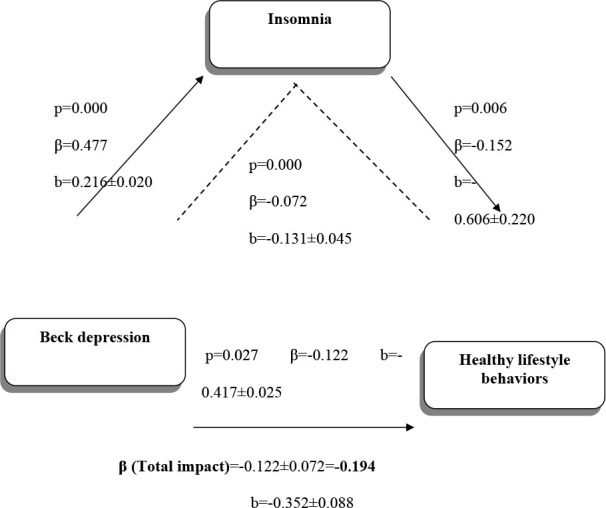
Research model.

Results reveal that both depression (β=-0.122) and insomnia (β=-0.152) have a significant negative effect on healthy lifestyle behaviors. According to another finding, insomnia has a mediating role in the effect of depression on healthy lifestyle behaviors (β=-0.072), and this role has a weak mediating effect. Insomnia further increases the negative effect of depression on healthy lifestyle behaviors (β=-0.194) (Figure 1).

## DISCUSSION

The menopausal phase emerges as a vulnerable period for the onset of depressive symptoms, with heightened stress levels often precipitating sleep disruptions^17-19^. In our study, both insomnia and depression were common among participating menopausal women, and a weak positive correlation was established between these two conditions. These findings resonate with similar studies that have elucidated the intricate relationship between sleep and depression^18-20^. Conversely, insomnia during this stage substantially elevates the risk of developing depressive symptoms by two to threefold^17^. On the contrary, Humeniuk et al. reported that menopausal women experienced insomnia and depression at rates similar to our study and that there was a negative relationship between them^20^. These different results suggest that insomnia and depression experienced by women may not only be related to menopause. The increase in conditions affecting women’s physical and mental health with advancing age may also cause these problems.

The findings of this study elucidate a positive correlation between symptoms of insomnia and depression, as well as a negative correlation with adherence to a healthy lifestyle. Essentially, this implies that both insomnia and depressive symptoms exert a substantial adverse influence on overall healthy lifestyle behaviors. Moreover, the study revealed that 50.9% of women experiencing pathological depression also reported instances of insomnia. These observations align with previous research positing a close association between sleep disorders and depression^8,19^. On the contrary, not having a healthy lifestyle and a good dietary routine can also be associated with insomnia and depression^21^.

Hachul et al. reported that older menopausal women experienced more severe insomnia and depression than younger women. The fact that the women who participated in our study entered menopause at a relatively young age may lead to more severe depression, sleep disturbance, and an unhealthy lifestyle in later ages if precautions are not taken^22^.

Among the women in this study, those who stated that they suffered from insomnia and whose sleep quality had deteriorated compared to the past, those whose daily sleep duration decreased (3–5 h/day), and those who experienced insomnia every day increased their susceptibility to depression and decreased their healthy lifestyle behaviors. Similar to our study, Baker et al. reported that 50% of menopausal women with sleep problems slept less than 6 h^2^. Insomnia and insufficient sleep experienced every day have a significant effect on the development and aggravation of depression^23^. During depression, the time to fall asleep increases, sleep depth decreases, sleep duration shortens, and frequent interruptions and early awakenings occur^23^. Not getting enough sleep at night often leads to daytime sleepiness, fatigue, depressed mood, difficulty in maintaining daily tasks, and other health and safety problems^24^.

Considering that the research is limited to women using social media in a certain part of Turkey, it is not possible to generalize the results to all women in the country. The dissemination of the surveys is only through the social networks of the researchers, and their circles represent a certain group. However, we believe that the inclusion of women spanning various educational backgrounds and age groups may reduce prejudices.

## CONCLUSION

The overall scores reflecting sleep quality positively correlated with depression, demonstrating an inverse association with adopting healthy lifestyle behaviors. Notably, our findings illuminate the mediating function of sleep issues in the impact of depression on the cultivation of a health-conscious lifestyle. This underscores the significant insight that the interplay between insomnia and depression during menopause notably influences the adoption of health-savvy practices. Specifically, more pronounced depressive symptoms are closely linked to heightened disruptions in sleep patterns. In order to augment awareness among middle-aged women regarding the multifaceted effects of menopause, it is strongly recommended to persist in conducting community-based studies.

## Data Availability

Data can be shared based on the reader’s reasonable request and priority base, and some restrictions will apply.
