# Erratum

**DOI:** 10.1590/1806-9282.20240943ERRATUM

**Published:** 2025-02-07

In the manuscript "The mediating role of insomnia in the effect of depression on healthy lifestyle behaviors in post-menopausal women", https://doi.org/10.1590/1806-9282.20240943, published in the Rev Assoc Med Bras. 2024;70(12):e20240943, on page 1:

**Where it reads:**

Review Article

**It should read:**

Original Article

**It was included:**

**ABSTRACT**

**OBJECTIVE:** This study aimed to ascertain the intermediary function of insomnia in the relationship between depression and healthy lifestyle behaviors in post-menopausal women.

**METHODS:** It included a cohort of 407 women. The data set was assessed using the Women's Health Initiative Insomnia Inventory, Beck Depression Inventory, and Healthy Lifestyle Behaviors Scale II. Then, the relationships between these variables were analyzed by correlation analysis, and for a more comprehensive study, the Statistical Package for the Social Sciences PROCESS macro Model 4 regression analysis was used.

**RESULTS:** Severe depression was detected in 42.5% of cases and insomnia in 35.1%. Sleep-related problems mediated the effect of depression with healthy lifestyle behaviors in a healthy context (β=0.072) and showed a weakly effective mediation effect (β=-0.194).

**CONCLUSION:** The study revealed that insufficient sleep intensified the adverse impact of depression on the adoption of healthy lifestyle practices.

**KEYWORDS:** Depression. Insomnia. Post-menopause. Mood. Sleep disorders.

